# Targeting Splicing in Prostate Cancer

**DOI:** 10.3390/ijms19051287

**Published:** 2018-04-25

**Authors:** Effrosyni Antonopoulou, Michael Ladomery

**Affiliations:** Faculty of Health and Applied Sciences, University of the West of England, Coldharbour Lane, Bristol BS16 1QY, UK; londinoantonopoulou@hotmail.com

**Keywords:** alternative splicing, prostate cancer, *VEGFA*, *KLF6*, *BCL2L2*, *ERG*, *AR*, splice switching oligonucleotides, RNA interference, splice factors, splice factor kinases

## Abstract

Over 95% of human genes are alternatively spliced, expressing splice isoforms that often exhibit antagonistic functions. We describe genes whose alternative splicing has been linked to prostate cancer; namely *VEGFA*, *KLF6*, *BCL2L2*, *ERG*, and *AR*. We discuss opportunities to develop novel therapies that target specific splice isoforms, or that target the machinery of splicing. Therapeutic approaches include the development of small molecule inhibitors of splice factor kinases, splice isoform specific siRNAs, and splice switching oligonucleotides.

## 1. Introduction

### 1.1. Pre-mRNA Splicing and Its Regulation

Pre-mRNA splicing was discovered in 1977 when the RNA sequence of adenoviruses was compared with their genome through the direct visualisation of RNA-DNA hybrids via electron microscopy. The hybrids of mRNA and restriction endonuclease cleavage fragments of viral DNA revealed loops that were unable to hybridize to the DNA. These intervening sequences were called ‘introns’ whereas the hybridized regions were called ‘exons’ [1,2].

Pre-mRNA splicing is catalysed by the spliceosome, a dynamic molecular machine which includes five small nuclear ribonucleoprotein particles (snRNPs) and a large number of auxiliary proteins [3]. These cooperate to recognize splice sites accurately and catalyse two transesterification reactions, each involving a nucleophilic attack on the terminal phosphodiester bonds of the intron [4]. Three sites participate in the splicing reaction: the 5′ splice site (5′ SS); the 3′ splice site (3′ SS); and the branch point sequence (BPS). It is now clear that the vast majority of human genes (>95%) are alternatively spliced, so that exons can be spliced together in different ways [5]. 

Pre-mRNA splicing is remarkably accurate thanks to the involvement of *cis*-regulatory elements that facilitate the identification of exon–intron junctions, preventing ‘pseudoexons’ from being included in mRNA. These elements can act as exonic splicing enhancers (ESEs) or silencers (ESSs), and intronic splicing enhancers (ISEs) or silencers (ISSs) [6,7]. They are recognised by ‘splice factors’, including RNA-binding proteins of the SR (serine/arginine-rich) family and the hnRNPs (heterogenous nuclear ribonucleoprotein) proteins [8]. SR proteins generally facilitate the recruitment of spliceosomal components such as the U1 snRNP to the 5′ splice site or U2AF65 to the pyrimidine tract adjacent to the 3′ splice site, facilitating recruitment of U2 snRNP across the branchpoint [9]. The SR protein SRSF1 binds to the ESE and facilitates splice site selection by promoting exon definition, helping to select alternative 5′ or 3′ splice sites in a concentration-dependent manner [10,11]. Increased concentrations of SRSF1 often promotes the selection of intron-proximal splice sites whereas increased concentrations of hnRNP A1 promotes that of intron-distal splice sites [12]. However, SR and hnRNP proteins can also do the opposite, depending on cell type [13,14].

Several splice factors, including SR proteins, undergo post-translational modifications which influence their interaction and specificity with other proteins as well as their local concentration adjacent to pre-mRNA substrates [15,16]. These modifications include phosphorylation by protein kinases such as AKT [17,18], DYRKs [19,20], SRPKs, and CLKs [21,22,23], and dephosphorylation by protein phosphatases including PP1 and PP2 [24]. These modifications also regulate SR protein contributions to the regulation of mRNA export [25], nonsense-mediated decay [26], and mRNA translation [27].

### 1.2. Extent of Alternative Splicing

In 1978, Wally Gilbert proposed that different combinations of exons could produce multiple mRNA isoforms of a single gene introducing the concept of alternative splicing [28,29]. Alternative splicing is the main source of proteomic and functional diversity and is crucial for the correct expression of the majority of metazoan genes [6,7]. A widely cited example that illustrates the ability of alternative splicing to generate a substantial repertoire of proteins is the *Drosophila melanogaster* gene *Dscam* (Down syndrome cell adhesion molecule). It can express 38,016 distinct splice isoforms from several clusters of mutually exclusive exons [30]. The isoforms differ in the three IgG-like domains in the extracellular portion of Dscam, changing their binding specificity. The different binding specificities affect interactions between cells, facilitating complex networks of neuronal connections. The neurexin genes are a well-studied family of genes in both invertebrates and vertebrates; they are required for the formation and function of synapses. They are highly diversified in vertebrates, and are also characterised by extensive alternative splicing through multiple mini-exons and alternative 5′ and 3′ splice sites producing an impressively wide range of splice isoforms [31]. Alternative splicing has been examined in the nematode model organism *Caenorhabditis elegans* demonstrating tissue-specific alternative splicing [32] and an involvement in sex determination [33]. Alternative splicing has also been documented in plants [34]; examples include alternative transcripts of *Vp-1* in wheat [35], and the *r1* gene [36] and the MADS box genes in maize [37].

There are several types of alternative splicing. The most prevalent type in vertebrates and invertebrates is exon skipping, in which whole exons are omitted from the mature transcript. Mutually exclusive exons are adjoining exons where only one is included in the mRNA. The boundaries and therefore sizes of exons can also vary through the use of alternative 5′ or alternative 3′ splice sites. Another form of alternative splicing is intron retention, in which individual introns are not removed. There are generally higher rates of intron retention in less complex metazoans [38]. The use of alternative exons can also arise due to alternative promoters or alternative polyadenylation sites. Nearly 95% of human genes are alternatively spliced expressing at least 90,000 isoforms from ~20,000 protein-coding genes [39]. Alternative splicing plays an important role in normal development and differentiation; aberrant alternative splicing is associated with an increasing range of human diseases including tauopathies, spinal muscular atrophy, retinitis pigmentosa, and many types of cancer [40,41]. Thus, understanding the nature and regulation of alternative splicing is a major topic in molecular biology nowadays. Its involvement in disease processes presents novel therapeutic opportunities.

## 2. Alternative Splicing and Prostate Cancer

### 2.1. The Challenge of Prostate Cancer

Prostate cancer (PCa) is one of the most important public health concerns worldwide with numbers of cases increasing significantly every year [42]. Its incidence is particularly high in western countries [43] where it is the most common malignancy and the second leading cause of death in men. In 2018, in the USA alone, the projected estimate is 29,430 deaths, 164,690 new cases [44] with an annual cost of treatment amounting to 12 billion dollars [45]. Lower incidence is observed in Asian countries; it is increasing in developed countries in South America, the Caribbean and sub-Saharan Africa [46]. These variations in incidence have been attributed, on the one hand, to diet, genetics, lifestyle, and environment and, on the other hand, to the availability of the prostate-specific antigen (PSA) screening test [43,47]. However, the PSA test lacks precision as elevated PSA levels are also associated with benign prostate lesions [48,49]. PCa is a highly heterogenous and multifocal disease ranging from relatively harmless, indolent disease to metastatic and lethal disease [50]. The biological diversity of PCa together with the development of resistance to androgen depletion therapies poses significant challenges in the clinic [51,52].

Alternative splicing contributes to tumour heterogeneity and is exploited by cancer cells, allowing them to divert away from normal developmental pathways [53]. It is therefore an appropriate time to look at alternative splicing as a possible context in which to develop novel targeted therapies for the treatment of PCa. In the following sections, we describe how alternative splicing affects genes that are clearly linked to the aetiology of PCa.

### 2.2. Vascular Endothelial Growth Factor (VEGFA)

Vascular endothelial growth factor VEGFA (also known as VEGF) is a key regulator of angiogenesis, required for cancer growth and metastasis [54]. VEGFA acts as a mitogen on endothelial cells through the VEGFR1, VEGFR2 and VEGFR3 receptors [55,56]. VEGFA is a member of a growth factor family with a common VEGF homology domain; the family includes VEGFA, VEGFB, VEGFC, VEGFD, VEGFE, VEGFF, and placental growth factors PIGF1 and -2 [57,58]. The conserved domain includes a cystine-knot structure formed by eight conserved cysteine residues [59].

The human VEGF genes generally contain seven exons; VEGFA has eight [60]. The VEGF genes are highly evolutionarily conserved [61]. At least 12 VEGFA isoforms are generated through alternative splicing of exons 6, 7 and 8 [62] giving rise to proteins with different heparin-binding properties [63]. VEGFA expresses both pro-angiogenic VEGF_xxx_ and anti-angiogenic VEGF_xxx_b isoforms, where xxx is the number of amino acids. The isoforms are generated through use of alternative 3′ splice site in exon 8; the proximal splice site results in pro-angiogenic VEGF and distal exon 8 splice sites anti-angiogenic VEGF [64]. VEGFA expresses at least seven additional splice isoforms due to alternative splicing of exons 6 and 7 generating VEGF_121_, VEGF_145_, VEGF_148_, VEGF_165_, VEGF_183_, VEGF_189_, and VEGF_206_, all of which exhibit different biological properties [58]. The anti-angiogenic isoforms are generally downregulated and the pro-angiogenic isoforms upregulated in tumours [65]. One of the main anti-angiogenic isoforms downregulated in a variety of cancers including PCa is VEGF_165_b [66]. Curiously, both alternative splice sites in exon 8 are followed by six amino acids before the stop codon; pro-angiogenic VEGFA ends in CDKPRR whereas anti-angiogenic VEGFA ends in SLTRKD [67]. This subtle change affects its interaction with the receptor VEGFR2 resulting in weakened downstream signalling [68]. The regulation of alternative splicing of VEGFA into pro- and anti-angiogenic isoforms has yet to be fully understood [69]. However, recent studies have identified that three SR proteins, SRSF6 [64,70], SRSF1 activated by the splice factor kinase SRPK1 [71], and SRSF2 [72] all contribute to its regulation. Pro- and anti-angiogenic VEGFA splice isoforms are not the only VEGFA splice isoforms that are biologically significant in prostate cancer. For example, an increase in the VEGF_121_/VEGF_165–189_ ratio in PC3 PCa cells results in a significant increase in angiogenesis in vivo, highlighting the importance of the VEGF_121_ isoform in PCa, and demonstrating that the balance of splice isoforms affects prostate carcinogenesis [73].

### 2.3. The KLF6 Gene

The *KLF6* gene product (Krüppel-like factor 6, also known as BCD1, COPEB, and ZF9) belongs to the family of Krüppel-like zinc finger transcription factors which consists of at least 24 members including Sp1-like (Sp1-8) and KFL-like factors (KFL1-16) [74]. It regulates a wide range of cellular processes including differentiation, proliferation, and apoptosis [75]. The gene is located on chromosome 10p15.2, a region deleted in about 55% of sporadic prostate adenocarcinomas [76]. It consists of four exons encoding a protein of 283 amino acids, including an N-terminus transactivation domain and three C-terminal C_2_–H_2_ zinc fingers that form a DNA-binding domain [77]. *KLF6* is a tumour suppressor gene, named after the gap gene Krüppel from *Drosophila melanogaster* that causes a crippled phenotype when inactivated [78]. It is down-regulated in several types of cancer including PCa through a range of mechanisms including loss of heterozygosity (LOH), somatic mutations, or aberrant alternative splicing [79,80].

The alternative splicing of *KLF6* results in the production of at least four splice isoforms [74]. One of the mutations found in PCa patients is a single nucleotide substitution in intron 1 that creates a novel binding site for the splicing factor SRSF5. This results in the use of two cryptic splice sites in exon 2 and enhances the expression of three alternative mRNA variants encoding for truncated KLF proteins; these are named Krüppel-like factor 6 splice variants 1, 2 and 3 (KLF6-SV1, SV2, and SV3). KLF6-SV does not have a zinc-finger DNA-binding domain but preserves most of the N-terminal domain [81]. KLF6-SV1 is formed by an alternative 5′ splice site in exon 3. A polymorphism of this isoform, IVS1 −27G>A, the *IVS*Δ*A* allele, was shown to be associated with increased risk of PCa when 142 probands from a familial PCa registry were examined [82]. KLF6-SV1 is an antagonist of *KLF6*, and acting in a dominant-negative manner, it can promote increased cell growth. Knockdown of full length *KLF6* increased tumour formation whereas knockdown of KLF-SV1 inhibited tumour formation [82]. Increased expression of KLF6-SV1 in tumours from men after prostatectomy is associated with lower survival rates and disease recurrence. When PCa cells are forced to overexpress this isoform in a mouse model, the mice are more susceptible to develop multi-organ metastases [83]. Increased expression of KLF6-SV1 is dependent on the Ras/PI3-K/Akt cell signalling pathway associated with increased cell proliferation and survival [84]. Together these findings suggest that the expression of the KLF6-SV1 isoform can promote metastatic phenotypes, and could be considered a target for therapy. The example also illustrates the point that a tumour suppressor gene can in fact acquire the properties of an oncogene, depending on how it is alternatively spliced.

### 2.4. The BCL2L1 Gene

The *BCL2L1* (BCL2-like 1) gene, commonly referred to as *BCLX,* is a member of the BCL-2 gene family. The BCL-2 family encodes part of the core of the apoptotic machinery and is conserved from the nematode *Caenorhabditis elegans* to mammals [85]. Apoptosis is a highly regulated process in development, and it is critical for the development and progression of cancer [86]. The BCL-2 family is composed of three groups; anti-apoptotic proteins, pro-apoptotic effectors and pro-apoptotic activators. They share at least one out of four conserved BCL-2 homology domains (BH1-4) [87]. *BCL2L1* has three exons and expresses several isoforms by alternative splicing with opposing functions, notably BCL-xL (B cell lymphoma extra-long) and BCL-xS (B cell lymphoma extra-small). The isoforms arise from alternative 5′ splice sites in exon 2. When the proximal splice site is selected, the BCL-xL isoform is expressed, whereas the BCL-xS isoform is expressed from the distal splice site [88].

The alternative splicing of *BCL2L1* is known to be regulated by several splice factors. The pro-apoptotic isoform BCL-xS is promoted by hnRNP H and F [89], SAM68 [90], RBM25 [91], and RBM11 [92]. In contrast BCL-xL is promoted by SRSF1 [93], SRSF9 [94], and SAP155 [95]. In PCa cells, treatment with the drug calyculin A blocks emetine-induced *BCL-xL* expression through inhibition of the protein phosphatases PP1 and PP2A, phosphatases that are known to dephosphorylate SR protein splice factors [96]. BCL-xS is down-regulated in many types of cancer including prostate cancer [97]. BCL-xL is associated with hormone refractory phenotype in PCa [98] and contributes to resistance to chemotherapy [99]. In contrast, BCL-xS inhibits cell survival [100] and the shift from BCL-xL to BCL-xS expression induces apoptosis in PC3 and MCF-7 cells [86]. There is therefore interest in developing therapeutic tools that alter the balance of BCL2L1 splice isoforms in tumour cells to promote apoptosis.

### 2.5. The ERG Gene

ERG (V-Ets avian erythroblastosis virus E26 oncogene homolog) is a member of the ETS (E26 transformation-specific) transcription factor family, one of the largest families of transcriptional regulators consisting of at least 27 members, subdivided into five subfamilies [101]. All ETS transcription factors contain a conserved ETS DNA-binding domain, consisting of 85 amino acids that recognize a core GGAA/T sequence [101]. ERG is expressed at low levels in normal prostate epithelial cells [102] and plays a key role in regulating a variety of cellular pathways such as cell proliferation, inflammation, apoptosis, and bone formation [103]. Strikingly, the *ERG* gene is highly expressed in more than 60% of patients with aggressive PCa [104].

*ERG* expression is generally activated through a 3 Mb deletion in chromosome 21 resulting in the fusion of the promoter of the androgen-regulated, prostate specific transmembrane serine protease *TMPRSS2* gene with the coding region of *ERG*, generating an androgen-responsive fusion oncoprotein [105]. Prostate cancer cells positive for the fusion show increased *ERG* expression in response to androgens due to the *TMPRSS2* promoter [106]. This fusion is the most common genetic lesion associated with disease mortality [107]. *TMPRSS2* can also fuse with the *ETV1* and *ETV4* genes (also members of the ETS family of transcription factors), but the fusion with ERG is the most prevalent, detected in ~50% of patients [108,109]. The *TMPRSS2-ERG* fusion is also associated with increased tumour growth and metastasis [110] and correlates with PSA recurrence and seminal vesicle invasion [111].

The *ERG* gene consists of 17 exons and expresses several splice isoforms of which at least five are translated; ERG-1, ERG-2, ERG-3, ERG-4, and ERG-5 [112,113]. *TMPRSS2-ERG* fusions express several fusion transcripts depending on the precise site of fusion, causing additional heterogeneity [114]. Recent evidence has underlined the biological relevance of cassette exon 7b of ERG. This exon encodes, in frame, 24 amino-acids that are added to the transcription activation domain in the middle of the protein. Rates of exon 7b inclusion increase in later stages of PCa, suggesting that it enhances ERG’s oncogenic potential [115], presumably by altering the effect of ERG on its target genes. Therapies could be designed that target, specifically, ERG’s cassette exon 7b.

### 2.6. The Androgen Receptor (AR)

The normal development and maintenance of the prostate is dependent on androgens whose production is regulated by the hypothalamic-pituitary gonadal through the androgen receptor (AR, also known as DHTR or NR3C4) [116]. AR is a 110 kDa member of the steroid receptor transcription factor family which includes the estrogen receptor-α (ERα), estrogen receptor-β (ERβ), and progesterone receptor (PR) [53]. Men express one copy of the *AR* gene located at Xq11-12. The gene has eight exons and the protein is 919 amino acids long with three major functional domains. These are the N-terminal domain, the DNA-binding domain and the ligand-binding domain each with a vital role in AR activity [117,118]. Alterations in AR transcripts including insertions of cryptic exons downstream of the coding sequences [119] or exon skipping [120] disrupt the open reading frame, leading to the expression of truncated proteins that are not androgen dependent. Reactivation of AR signalling contributes to castration-resistant PCa [121]. There is still a degree controversy in relation to the association between AR activation and disease progression; some studies suggest that high AR expression increases tumour grade and stage leading to lower recurrence-free survival [122], whereas other studies associate decreased AR expression with tumour progression [123]. These discrepancies could be explained by the fact that it is not just overall expression of AR that matters, but also its alternative splicing.

There are at least 22 splice variants of AR [124] with AR-V7 and AR^V567es^ the most commonly studied; of these, AR-V7 has been detected in the clinic [125]. Both splice variants lack the C-terminal ligand-binding domain; they are constitutively active and therefore resistant to traditional androgen deprivation therapy [126]. AR-V7 mRNA arises from an alternative 3′ splice site next to a cryptic exon 3B [127]. The detection of AR-V7 transcripts in circulating tumour cells of 62 men with progressive, metastatic castration-resistant PCa was associated with resistance to the AR inhibitors abiraterone and enzalutamide [128]. The splice factors U2AF65 and SRSF1 have been found to play a critical role in mediating AR-V7 expression [129].

AR^V567es^ arises from exon 5 to 7 skipping causing an early stop codon after the first 29 nucleotides of exon 8 [130]. AR^v567es^ has also been found to activate androgen-responsive genes such as *KLK3*, *TMPRSS2*, and *NKX3.1* in a hormone-independent manner when ectopically expressed in LNCaP cells [131]. Using a *probasin* promoter driven AR^v567es^ transgenic mouse, it was found that expression of AR^v567es^ in prostate leads to epithelial hyperplasia after 16 weeks, and invasive adenocarcinoma by year one in the experimental model. However, the precise role of the AR^v567es^ in normal prostate growth and castration-resistant PCa is still unclear [132].

Additional splice variants of AR have been described. One was identified by RACE (rapid amplification of cDNA ends) in CWR-R1 cells and named AR8. Its expression is highest in cells derived from castration-resistant tumours [133]. AR8 lacks a DNA-binding domain and localizes to the plasma membrane. Overexpression of AR8 promotes the association of Src and AR with the EGF receptor following EGF treatment. Another study described AR45, an isoform that lacks exon 1. Its overexpression decreases the proliferation of androgen-dependent LNCaP cells through the formation of AR-AR45 heterodimers, presumably by inhibiting the activity of full length AR [134]. In summary there are several, functionally distinct AR splice variants that contribute to androgen resistance mechanisms. It is therefore imperative to develop novel therapies that modify AR alternative splicing.

## 3. Modulation of Splicing for Therapeutic Benefit

Clinically significant splice isoforms often exhibit unique biological properties and can be targeted by pharmacological agents directly, or through targeting the splice factors that regulate their expression [135]. To illustrate this point, small molecular inhibitors of the SRPK1 splice factor kinase that phosphorylates the oncogenic splice factor SRSF1 can switch VEGFA splicing in favour of the anti-angiogenic isoforms in PCa cells [71]. SRPK1 inhibitors decrease tumour growth in a PCa mouse model in vivo providing strong evidence that they could be used therapeutically for treating PCa [72,136,137]. SPHINX is the latest generation SRPK1 inhibitor; it exhibits anti-tumour effects after repetitive intraperitoneal administration in a mouse model of orthotopic PCa [136]. The inhibition of SRPK1 brings benefits to other pathologies that involve abnormal angiogenesis. For example, a single dose of an earlier SRPK1 inhibitor, SRPIN340, significantly inhibits angiogenesis and increases normal vascularization in a mouse model of retinal neovascularization [72].

Small molecule inhibitors can also be used to alter the expression of AR splice isoforms through the inhibition of HSP90, a protein required for nuclear translocation of full-length AR in response to hormone. The first HSP90 inhibitor in clinical trials is alvespimycin [138]; it produces a good response in castrate-refractory PCa, and a stable response in patients with melanoma, chondrosarcoma, and renal cancer [139]. A second-generation HSP90 inhibitor called onalespib inhibits more than 500 splice isoforms including AR splice isoforms. In particular, onalespib was found to reduce the levels of both full length AR and AR-V7 in PCa cell lines in a concentration and time-dependent manner; it also reduced tumour growth in AR-V7 expressing 22Rv1 tumour xenografts [140]. However, the effect of HSP90 inhibition on the expression of AR splice isoforms is not yet entirely clear. Treatment with the second-generation HSP90 inhibitor luminespib increased AR-V7 expression in VCaP cells [141] whereas geldanamycin did not affect AR-V7 levels in 22Rv1 cells [142].

Another approach is the use of splice-switching oligonucleotides (SSOs). SSOs are short (15–25 bases in length), synthetic, antisense, modified nucleic acids that target exon-intron junctions or regulatory exonic and intronic sequences [143]. Their binding leads to the blocking of the RNA–RNA base-pairing or protein–RNA binding interactions that occur between components of the splicing machinery and the pre-mRNA [144]. SSOs have been used to modify splicing of *BCL2L1*, disrupting tumour growth in vivo by targeting the 5′ splice site of exon 2 of *BCL2L1* pre-mRNA. This led to decreased expression of Bcl-xL and an increased expression of Bcl-xS splice isoform, inducing apoptosis in breast and PCa cells and reducing the tumour burden in vivo [145]. Another study found that the Bcl-xS isoform, induced by SSOs, sensitized the cancer cells to treatment with ultraviolet, γ-irradiation and chemotherapeutic drugs [99].

Small interference RNAs (siRNAs) have been widely used to target splice variants and counteract their oncogenic activities. SiRNAs have been used to target specific AR exons, for example exon 1 in human prostate carcinoma 22Rv1 cells reduced cell proliferation [146]. SiRNA-mediated knockdown of AR isoforms that lack the C-terminal hormone binding domain suppressed androgen-independent cell proliferation, and induced G1 cell-cycle arrest and apoptosis; similar results were obtained through treatment with the antibiotic nigericin, known to act at a post-transcriptional level on AR expression [147].

**Figure 1:**
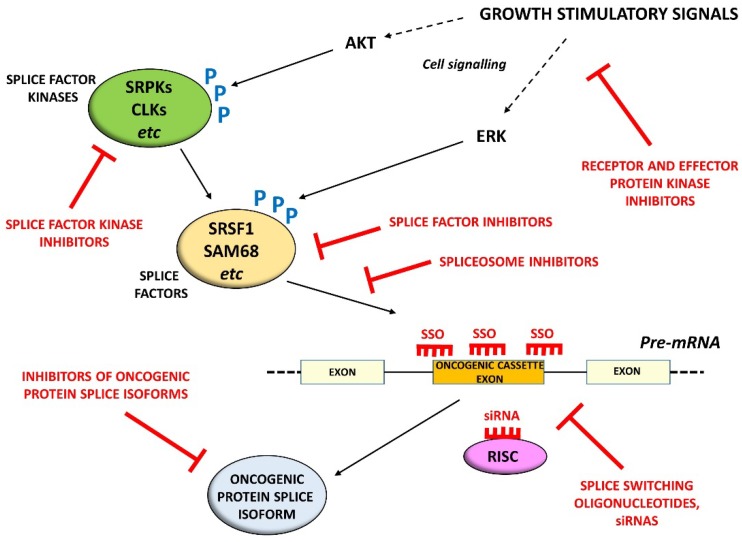
Different approaches to the in vivo modification of splicing of cancer-specific isoforms. Growth stimulatory signals that activate splice factor kinases through AKT or splice factors directly through ERK can be inhibited; splice factor kinases, splice factors or components of the spliceosome can also be targeted directly. At the RNA level, pre-mRNA splicing can be modified with splice switching oligonucleotides (SSOs), and specific mRNA isoforms can be targeted with siRNAs. At the protein level, drugs can also be targeted at specific isoforms.

Figure 1 summarizes different approaches to altering splicing in vivo. Splice factor kinases [71,136], splice factors [148], or even spliceosome components [149] can be targeted directly. RNA interference-based methods [150,151] and SSOs [86,152] have been successfully used to target specific splice isoforms at the RNA level. It seems highly likely that prostate cancer patients will benefit in the future from therapies directed at altering splicing of cancer-associated genes. The challenge will be to improve the stability, specificity, and delivery of modifiers of splicing to tumour sites, while minimising side-effects [152].

## 4. Conclusions

It is abundantly clear that alternative splicing is vital for normal gene regulation, and that aberrant splicing is involved in many types of cancer, including prostate cancer. Therefore understanding the regulation of alternative splicing can provide new contexts for the development of novel therapeutic strategies. A number of cancer specific splice variants have been identified and discussed in this article, including *VEGFA*, *KLF6*, *BCL2L2*, *ERG,* and *AR*. Novel therapeutic strategies including the use of SSOs to alter the balance of pro- and anti-apoptotic BCL2L2 isoforms, siRNAs that target specific AR exons, small molecule inhibitors that block AR-mediated signalling, or small molecules that inhibit splice factor kinases, have all shown promise. There is a pressing need to expand research into alternative splicing not just in the context prostate cancer, but in the context of many other important diseases.

## Abbreviations

BPS	branchpoint sequence
ESE	exonic splice enhancer
ESS	exonic splice silencer
ISE	intronic splice enhancer
ISS	intronic splice silencer
hnRNP	heterogenous ribonucleoprotein
RACE	rapid amplification of cDNA ends
snRNP	small nuclear ribonucleoprotein
SR protein	serine/arginine-rich splice factor
SSO	splice-switching oligonucleotide
PCa	prostate cancer
